# Affective Processing in Non-invasive Brain Stimulation Over Prefrontal Cortex

**DOI:** 10.3389/fnhum.2017.00439

**Published:** 2017-09-04

**Authors:** Wei Liu, Ya Shu Leng, Xiao Han Zou, Zi Qian Cheng, Wei Yang, Bing Jin Li

**Affiliations:** ^1^Jilin Provincial Key Laboratory on Molecular and Chemical Genetic, The Second Hospital of Jilin University Changchun, China; ^2^Department of Anesthesiology, The Third Hospital of Jilin University Changchun, China

**Keywords:** NBS, TMS, tDCS, tACS, affective processing, PFC

## Abstract

The prefrontal cortex (PFC) is the most frequently targeted brain region by non-invasive brain stimulation (NBS) studies. Non-invasively stimulating the PFC has been shown to both modulate affective processing and improve the clinical symptoms of several psychiatric disorders, such as depression and schizophrenia. The magnitude of the modulation depends on several factors, including the stimulation frequency, the number of stimulation sessions, and the specific sub-region of the PFC that is stimulated. Although some of the potential underlying mechanisms have been identified, the exact mechanisms that underlie these cognitive and affective changes remain unclear. The present review aims to summarize recent advances in the study of affective processing using NBS over the PFC. We will provide a theoretical framework for better understanding how affective processing changes are induced by NBS, with the goal of providing testable hypotheses for future studies.

## Introduction

Affective processing plays a significant role in survival and adaptation functions and often interacts with other cognitive processes such as memory, learning, decision making, motivation, and reactions to environmental circumstances (Davidson, 2002). Several biobehavioral scientists share the idea that there are two fundamental affective systems: those relating to positive and those relating to negative effects (Baker et al., 2014). Although the two systems rely on distinct neuroanatomical circuitry, increasing evidence suggests that the prefrontal cortex (PFC) and amygdala are involved in both systems (Pessoa and Adolphs, 2010; Li et al., 2017). Affective processing deficits are often a core factor in most psychiatric disorders such as depression, schizophrenia, phobia, anxiety disorder, bipolar disorder, and addiction. Non-invasive brain stimulation (NBS), which includes techniques such as transcranial magnetic stimulation (TMS) and transcranial direct/alternating current stimulation (tDCS/tACS), have been shown to be effective neuromodulating therapies for psychiatric disorders where impaired affective processing is a core feature, and have consequently shown great potential for future clinical applications. The PFC is the most frequently targeted region by NBS. To our knowledge, in contrast to the direct stimulation of the PFC, the effects of NBS on the amygdala are predominantly achieved through non-invasive stimulation of the PFC.

## Affective Processing of TMS over the PFC

Transcranial magnetic stimulation is developed on the basis of electromagnetic induction. The change of the extrinsic magnetic pulses on brain can produce an ionic current in the brain which can preferentially activate the pyramidal cells or at their axons and induce the change of neural excitability (Kobayashi and Pascual-Leone, 2003). Transcranial magnetic stimulation involves three modes: single pulse TMS, pair-pulse TMS, and repetitive TMS (rTMS). Repetitive TMS is the most mode in the therapy of depression and other psychiatric disorders. The Food and Drug Administration (FDA) has approved rTMS as an effective antidepressant treatment for medication-resistant patients with major depression. In clinical trials of psychiatric disorders, the PFC is the most studied brain regions with rTMS. The PFC is an important neural hub involved in mood and affective processing, in addition to playing an important role in the attentional control of affective information. The clinical effects of low-frequency (1 Hz) rTMS over the PFC are limited and significantly less than high-frequency (>1 Hz) rTMS (HF-rTMS) over the PFC (Alonso et al., 2001; Cohen et al., 2004). Therefore, most of the studies of rTMS are based on HF-rTMS. High-frequency rTMS is known to induce a depolarization of the neurons in the PFC (Guse et al., 2010). Its application over the dorsolateral PFC (dlPFC) was shown to produce a greater cognitive improvement in patients with psychiatric/neurological diseases than in healthy participants (Guse et al., 2010). The exact underlying neural mechanisms responsible for this improvement remain unknown. Besides, the number of stimulation sessions is likely to play a role, as a single session of HF-rTMS applied over the dlPFC was not sufficient to produce significant mood changes in either normal volunteers or depressed patients (Baeken et al., 2008; Leyman et al., 2011).

## Affective Processing of TMS over the PFC in Depression

Affective processing of rTMS over the PFC has been shown in some affective disorders, such as depression, schizophrenia, and obsessive compulsive disorder. Depression is the most thoroughly studied in the clinical trials of rTMS. Repetitive TMS, as a typical mode of antidepressant treatment, has shown favorable effects on the depression. Although many studies have investigated the neural mechanisms of TMS over the PFC in depression, however, results are not always consistent and a clear picture has yet to emerge. High-frequency rTMS applied over the left dlPFC protects against the development of negative mood symptoms and increases the susceptibility to negative emotional stimuli (Möbius et al., 2016). High-frequency rTMS (10 Hz) over the left dlPFC of the depressed patients can improve the overall mood status, bias attentional processing toward negative information, and improve the Hamilton depression rating scale (Leyman et al., 2011). Although the first HF-rTMS stimulation session had no effect on the participants, there was a significant improvement following a 2-week treatment. However, no evidence showed the optimal treatment duration. The application of rTMS over the dlPFC also has effects on the cognitive control network and affects associated with cognitive control processes (Lantrip et al., 2017). However, measures of decision-making and impulse control were not affected by the application of rTMS over the left dlPFC, though measures of depression and anxiety both showed significant improvement (Tovar-Perdomo et al., 2017). This finding suggests that neurocognition may not be related to the psychopathological symptoms observed in these mood disorders. Furthermore, different rTMS stimulation frequency rates when applied over the dlPFC may have differential mood effects in depressed patients (Kimbrell et al., 1999; Speer et al., 2000; Speer et al., 2009). Although both low-frequency (1 Hz) and HF (20 Hz)-rTMS have shown to produce positive results, depressed patients who display therapeutic improvements for one stimulation frequency may actually get worse when using other stimulation frequencies (Kimbrell et al., 1999). After 2 days of rTMS treatment, a widespread increase in cerebral blood flow was observed following high-frequency (20 Hz) stimulation, whereas low-frequency (1 Hz) stimulation produced a decrease in cerebral blood flow (Speer et al., 2009). Moreover, depressed patients with lower baseline metabolism levels showed a better response to high-frequency (20 Hz) stimulation, whereas those with higher baseline levels showed a better response to low-frequency (1 Hz) stimulation (Speer et al., 2000, 2009). In contrast, other studies have reported that both 5 Hz-rTMS and 10 Hz-rTMS may produce similar clinical effects on patients with major depressive disorder (MDD; Philip et al., 2015).

Several studies have demonstrated that important dlPFC hemispheric differences exist in MDD: the left dlPFC tends to be hypoactive, whereas the right dlPFC is generally hyperactive (Maeda et al., 2000; Mayberg, 2003; Phillips et al., 2003; Fahim et al., 2004; Bermpohl et al., 2006; Grimm et al., 2008; Lally et al., 2015). This finding suggests that the left and right dlPFC might serve distinct functions and be differently affected in MDD. The hypoactivity in the left dlPFC is related to emotional judgment and the modulation of positive emotional valence in MDD, whereas the hyperactivity in the right dlPFC is associated with attention to emotional judgment and depression severity in MDD (Grimm et al., 2008). Hyperactivation of the right dlPFC can also induce a consistent attentional bias toward negative and aversive memories.

The activation of the left dlPFC caused by rTMS treatment can improve the memory retrieval of positive affective memories, whereas symptoms of anxiety can be induced by the stimulation of the right dlPFC (Balconi and Ferrari, 2012). The application of rTMS over the left dlPFC produces an increased neuronal excitability in the left dlPFC, and recovered the normal responsivity to emotional judgment. It may be a mechanism of recovery in MDD. Besides, the application of rTMS over the left dlPFC can also increase brain activity in the left dlPFC and the anterior cingulate gyrus, whereas it has been shown to decrease brain activity in the left fusiform gyrus, the left cerebellum, and the right dlPFC (Cardoso et al., 2008). It was also shown that rTMS produced a similar clinical improvement in depressed patients when compared with a fluoxetine treatment. Another common clinical symptom observed in depressed patients is an inability to shift their attention focus, an impairment that is generally attributed to a dysfunction in the dlPFC. A single session of HF-rTMS over the left dlPFC can benefit task-switching performance and stabilize the patient’s mood (Vanderhasselt et al., 2009). Although a single session of HF-rTMS over the left dlPFC is insufficient to produce subjective mood changes, it can significantly affect the hypothalamic–pituitary–adrenal (HPA)-axis by reducing salivary cortisol levels both immediately and 30 min after the stimulation (Baeken et al., 2009). These results indicate that changes involving the endocrine system may underlie the affective processing improvements.

The right dlPFC is related to symptoms of anxiety, depression, and panic. Chronic low-frequency rTMS over the right dlPFC showed a significant improvement in panic symptoms after 4 weeks of treatment, with the improvement peaking approximately after 8 weeks. However, the improvement of depressive symptoms required a longer treatment duration, where approximately only half the patients showed improvements after 8 weeks of treatment (Mantovani et al., 2013). The application of rTMS treatment to the right dlPFC can also increase subsequent event-related neural activity observed in bilateral, parietal, and temporal cortex; affect early stage affective visual processing; and enhance differential affective responses to fearful stimuli in individuals with anxiety (Zwanzger et al., 2014). Additionally, the right dlPFC may also be involved in the attentional processing of emotional information.

Multiple rTMS sessions applied to prefrontal regions may modulate the cortical excitability and improve the attentional control over emotional information. However, a single HF-rTMS session applied over the right dlPFC was shown to immediately impair the ability to inhibit negative information, whereas no significant changes were found when the left dlPFC was stimulated (Leyman et al., 2009). Such hemispheric stimulation differences were not observed for self-rated mood measures and emotionally induced facial expressions following dlPFC stimulation (Luo et al., 2010). Although both the left and right dlPFC play an important role in affective processing, sequential bilateral rTMS does not provide an added benefit compared with the application of HF-rTMS over the left dlPFC alone (Fitzgerald et al., 2012, 2016).

Besides the change of neural excitability, the alteration of various neurochemicals also contributes to neurobiological mechanisms of the antidepressant effect of rTMS. Repetitive TMS has been shown to change the endocrinological serum levels in the brain of healthy subjects (Evers et al., 2001). Infra-threshold stimulation only mildly decreased cortisol and thyroid stimulating hormone (TSH) serum levels in both the males and females. However, although this effect might stem from increased prolactin production in females, effects on other hormones were not observed. High-frequency rTMS (25 Hz) stimulations over the left dlPFC can decrease brain-derived neurotrophic factor (BDNF) serum levels in healthy men (Schaller et al., 2014). This effect can be further modulated by the smoking status of the male volunteers. It remains unknown whether sex is an additional modulating factor. However, the reported changes in BDNF levels have been inconsistent. Indeed, the findings of other studies have suggested that the use of acute rTMS over the PFC in healthy volunteers does not produce significant BDNF serum level changes (Lang et al., 2008). Moreover, rTMS applied over the dlPFC can also affect the cerebral glucose metabolism levels. In patients with medication-resistant depression, there are lower glucose metabolism that tends to be lower bilaterally in the dlPFC and in the anterior cingulum, whereas in contrast, several limbic and subcortical regions show increased glucose metabolism levels. After 2 weeks of rTMS treatment, however, glucose metabolism levels in the left middle temporal cortex and the fusiform gyrus significantly decreased in the patients who showed a better response; the general cerebral glucose metabolism pattern remained abnormal, however (Li et al., 2010). Finally, some animal studies and healthy human studies have shown that acute rTMS over the left dlPFC can transiently increase dopamine levels, whereas chronic rTMS does not produce the same effect (Kuroda et al., 2006).

The dorsomedial prefrontal cortex (dmPFC) is another important brain region that is affected by depression. Gray matter density of the dmPFC is reduced in depression, and the magnitude of this reduction correlates with the severity of depression (Koenigs et al., 2008; Koenigs and Grafman, 2009). Transcranial magnetic stimulation applied over the dmPFC can significantly improve the severity of clinical symptoms of depression by increasing the connectivity between the dmPFC and the thalamus, and also between the subgenual cingulate cortex and the caudate nucleus (Salomons et al., 2014; Dunlop et al., 2015; Schulze et al., 2016).

## Affective Processing of TMS over the PFC in other Disorders

Transcranial magnetic stimulation is also increasingly considered as a promising treatment for schizophrenia. Active HF-rTMS (10 Hz) applied to the left dlPFC can induce a significant improvement of the negative clinical symptoms of schizophrenia and associated emotional functioning (Prikryl et al., 2013). These effects may be attributed to both an increase in neuronal activity and an increase of neurotransmitters or receptors. Stimulation over the left dlPFC may also enhance the neural connectivity between the PFC and the amygdala. However, high-frequency bilateral rTMS did not result in any substantial benefit regarding the negative symptoms of schizophrenia (Fitzgerald et al., 2008). The mechanism remains unknown. Deep rTMS applied over bilateral PFC also can significantly reduce self-oriented anxiety during difficult and affective social situations in patients with autism spectrum disorder (Enticott et al., 2014). Furthermore, rTMS applied over the left dlPFC can improve impaired affective processing in patients with obsessive compulsive disorder (de Wit et al., 2015). Potential underlying mechanisms include a reduction in neural activity within the left dlPFC and an increase in connectivity between frontal areas and the amygdala. In addition, rTMS over PFC also significantly decreased drug cue craving in drug-addicted patients, including heroin and cocaine, and no significant adverse events were noted (Shen et al., 2016; Terraneo et al., 2016).

## Affective Processing of tDCS over the PFC

Transcranial direct current stimulation is another non-invasive brain neuromodulation technique that is receiving more attention within the research community. In tDCS, a constant and low intensity direct current (1–2 mA) is delivered to the brain area via electrodes on the scalp. Direct current can influence the frequency of neuronal spontaneous discharge. The frequency gets higher when the anode of tDCS closes to the soma and dendrite of neurons, while the cathode of tDCS makes it lower (Wagner et al., 2007). Besides, tDCS can only affect the active neurons.

Transcranial direct current stimulation may affect cortical neuronal activity in the dlPFC, and the tDCS polarity (cathodal or anodal) applied to the dlPFC modulates the performance of participants during a stimulus–response compatibility task (Morgan et al., 2014). Cathodal current inhibits cortical excitability, whereas anodal current enhances it (Da et al., 2013). Transcranial direct current stimulation applied over the dlPFC had no influence, however, on self-reported affective state measures in healthy individuals (Morgan et al., 2014). However, the result was not consistent with the other studies in healthy volunteers. Polarity of tDCS to the left dlPFC had shown converse results in affective processing. Although some studies found that anodal tDCS applied to the left dlPFC decreased negative mood valence both during and after stimulation in healthy participants, other studies reported the opposite finding (Boggio et al., 2009; Peña-Gómez et al., 2011; Maeoka et al., 2012). Cathodal tDCS applied to the left dlPFC has been shown to have no effects on negative mood (Peña-Gómez et al., 2011; Nitsche et al., 2012). Moreover, tDCS has been shown to produce different physiological effects when applied to the left compared with the right dlPFC. Anodal tDCS applied to the right dlPFC (or cathodal tDCS to the left dlPFC) can significantly regulate affective impulses, whereas the opposite stimulation conditions can promote risky decision-making behavior (Pripfl et al., 2013).

The effects of tDCS on depression, schizophrenia, substance use disorders, obsessive compulsive disorder, generalized anxiety disorder, and anorexia nervosa have been extensively studied (Khedr et al., 2014; Kekic et al., 2016; Lefaucheur et al., 2017). Although some studies have reported its effectiveness for psychiatric disorders, generalizations should be made with caution given that the tDCS technique is still in its infancy compared to other brain stimulation techniques.

## Affective Processing of tDCS over the PFC in Depression

Transcranial direct current stimulation has been demonstrated as an effective, relatively safe and convenient treatment option for depression, which has been the most extensively studied clinical condition with this neuromodulation technique. The most studied brain region with tDCS in depression patients is the dlPFC, which plays a significant role in modulating emotions and motor cortex excitability. Cathodal stimulation current inhibits cortical excitability, whereas anodal current enhances it (Da et al., 2013; Brandão et al., 2015). The European Chapter of the International Federation of Clinical Neurophysiology produced guidelines that recommended the application of anodal tDCS over the left dlPFC (with a right orbitofrontal cathode) as a treatment option for patients with a MDD with (C level recommendation) and without drug resistance (B level recommendation) (Lefaucheur et al., 2017).

There are two neural networks involved in the neurobiology of depression. The first network underlies “hot” cognitive processes, is implicated in affective and reward processing, and is subserved by the limbic structures and the ventral PFC. The second network underlies “cold” cognitive processes; is related to non-emotional cognitive processing; and is subserved by the dorsal anterior cingulate cortex, the hippocampus, and the dlPFC (Drevets et al., 2008; Roiser and Sahakian, 2013; Nord et al., 2017). Cognitive deficits and executive dysfunctions are signature characteristics of major depression, and have been associated with the emotional dysregulation observed depression patients (Salehinejad et al., 2017). Ten sessions of tDCS applied over the dlPFC can significantly improve both the depressive symptoms and cognitive functions—such as working memory and attention—that are affected by depression. Working memory deficits are a common symptom associated with depression and other psychological conditions, such as schizophrenia. Working memory deficits can also be observed in healthy individuals exposed to situations producing acute stress. The application of anodal tDCS over the dlPFC can improve stress-induced working memory deficits in depression patients (Bogdanov and Schwabe, 2016). Furthermore, tDCS can enhance the improvement of working memory induced by neurocognitive training in depression patients (Vanderhasselt et al., 2015). Transcranial direct current stimulation can also modulate negative attentional bias measures in as little as a single tDCS session (Brunoni et al., 2014). The combined treatment of cognitive control training and tDCS applied over the dlPFC has been shown to produce a better improvement for depressed patients compared with tDCS alone (Segrave et al., 2014).

Transcranial direct current stimulation can enhance neuronal activation in the PFC, dorsal hippocampus, ventral tegmental area, and nucleus accumbens (Peanlikhit et al., 2017). Transcranial direct current stimulation also enhances vagal activation, inhibits the HPA activity, and decreases the salivary cortisol levels (Brunoni et al., 2013b). Unlike TMS, tDCS may have no significant influence on the BDNF levels in depression patients (Palm et al., 2013).

A recent study indicated that anodal tDCS stimulation of the left dlPFC (or cathodal stimulation of the right tDCS) reduced vigilance levels in response to threat-related affective stimuli in healthy individuals (Ironside et al., 2016). The authors of this study also put forward the idea that the processing of threat-related affective information was a sensitive evaluation criterion to determine treatment effectiveness in clinical settings (Ironside et al., 2016). Finally, it has also been shown that anodal tDCS reduces cortisol levels and enhances vagal activity, both of which are involved in stress regulation (Brunoni et al., 2013b).

Although there is no mechanism to reveal the interactions between tDCS with antipsychotics and nonbenzodiazepine anticonvulsants in depressed patients, some antipsychotics, such as SNRIs, SSRIs, and tricyclics, can significantly enhance the improvement of tDCS (Brunoni et al., 2013a). However, benzodiazepine can reduce the improvement associated with tDCS (Brunoni et al., 2013a). The use of a tDCS treatment after the administration of lorazepam (a GABAergic agonist) can initially reduce cortical excitability only to later increase excitability (Nitsche et al., 2004). Anodal stimulation has been shown to decrease local gamma-aminobutyric acid (GABA) levels, whereas cathodal stimulation inhibits glutamatergic activity (Stagg et al., 2009). GABA/glutamate levels are also modulated by benzodiazepine, which may explain the interaction effects observed when combining tDCS with benzodiazepines (Hasan et al., 2012).

## Affective Processing of tDCS over the PFC in Schizophrenia

Transcranial direct current stimulation applied to the PFC has been shown to be an effective and relatively safe therapeutic option for adults and children with schizophrenia (Mattai et al., 2011). Negative symptoms and cognitive dysfunction are important facets of schizophrenia and are under the regulation of the fronto–thalamic–parietal and frontal–striatal networks (Galderisi et al., 2015; Palm et al., 2016). The dlPFC is a central hub in both of these networks and is functionally impaired in schizophrenia, leading to important cognitive deficits (Galderisi et al., 2015; Sheffield et al., 2015). Negative symptoms include a flattening of the affect, alogia, avolition–apathy, anhedonia–asociality, and attention disorders (Palm et al., 2016). Furthermore, the currently available evidence suggests that negative symptoms and cognitive dysfunctions are both associated with the affective processing deficits observed in schizophrenia. The application of prefrontal tDCS combined with stable antipsychotic medication has been shown to improve negative symptoms in patients with severe schizophrenia (Palm et al., 2016).

Prefrontal tDCS has also been shown to improve working memory performance and affective identification performance in patients with schizophrenia, and to enhance connectivity between brain networks in healthy individuals as measured by resting-state functional connectivity magnetic resonance imaging (fcMRI) (Rassovsky et al., 2015; Orlov et al., 2017). Auditory verbal hallucinations constitute another important clinical symptom in schizophrenia and are believed to be related to abnormal hyperactivity in the left temporoparietal junction, in addition to abnormal connectivity between frontal and temporal brain areas. The application of tDCS over the left dlPFC can significantly reduce the occurrence of auditory verbal hallucinations through the regulation of resting-state functional connectivity between brain areas in the related network (Mulquiney et al., 2011; Andrade, 2013; Mondino et al., 2016).

## Affective Processing of tDCS over the PFC in Drug Dependence and Bulimia Nervosa

The PFC also plays a critical role in developing addictions, particularly those related to drug use and smoking. Drug dependence has been associated with a reduction of brain activity within the PFC, which is believed to be subsequent to structural and functional damage to the PFC (Goldstein and Volkow, 2002; Da et al., 2013). Cravings are associated with enhanced drug cue-related prefrontal activation (Tapert et al., 2003; Wilson et al., 2004; McBride et al., 2006). The application of tDCS over the left dlPFC is reported to inhibit the neural activation within the PFC normally triggered by drug-related cues, and to reduce acute substance cravings in drug addicts (Da et al., 2013; Wang et al., 2016). Moreover, tDCS can improve the general mood status of drug addict patients (Da et al., 2013).

Bulimia nervosa is an abnormal and excessive food craving disorder that can lead to other health problems including obesity. Some studies have indicated that the application of tDCS over either the right or the left dlPFC can enhance inhibitory control while reducing food cravings and intake (Burgess et al., 2016; Ljubisavljevic et al., 2016; Macedo et al., 2016; Georgii et al., 2017; Kekic et al., 2017; Lowe et al., 2017). The specific underlying mechanisms may involve an increased regulation of the reward and decision-making neural networks (Fregni et al., 2008).

## Affective Processing of tDCS over the PFC in other Disorders

Posttraumatic stress disorder produces deficient fear extinction mechanisms and is associated with an abnormal activation of key brain regions that are part of the fear extinction network, which includes the ventromedial PFC, amygdala, and hippocampus (Marin et al., 2014). Furthermore, the integrity of this network is related to symptom severity in posttraumatic stress disorder. Transcranial direct current stimulation has been shown to enhance the recall of positive memories that improve fear extinction mechanism in posttraumatic stress disorder (Penolazzi et al., 2010; Asthana et al., 2013; Marin et al., 2014).

Pain is a multidimensional experience involving cognitive, attentional, and affective processes. Pain is often associated with negative emotions such as unpleasantness or anxiety, which can, in turn, modulate the pain experience. The application of tDCS over the left dlPFC can alleviate affective pain by increasing the neural activity in the left dlPFC, and also by down-regulating pain inhibitory systems (Maeoka et al., 2012). This mechanism may rely on a distinct and independent pathway from those regulating the somatosensory perception of pain (Boggio et al., 2009).

## Affective Processing in tACS over the PFC

There are only a few studies that have investigated the effects of tACS on affective processing. These studies focused primarily on improving working memory functions in both healthy individuals and patients with schizophrenia (Meiron and Lavidor, 2014; Hoy et al., 2016). It has also been suggested that the bi-frontal application of tACS might improve functional connectivity between the different prefrontal regulatory components of working memory in humans. Furthermore, tACS applied to the left dlPFC can entrain endogenous cortical oscillations and produce working memory improvements in schizophrenia patients similar to those observed following tDCS.

## Discussion

Affective processing plays a significant role in survival and adaptation functions. Disturbance of affective processing is a core factor for most psychiatric disorders, such as, anxiety, depression, schizophrenia, phobia, and addiction. Although NBS studies have provided tantalizing symptom improvement and affective processing in some psychiatric disorders, such as depression and schizophrenia, there are still lots of limits for clinical applications. As we reviewed, depression is the most thoroughly studied affective disorder in TMS and tDCS; however, there are no standardized treatment protocols in the clinical application of rTMS for depressived patients. Repetitive TMS over the left dlPFC can improve the overall mood status, bias attentional processing toward negative information, increase susceptibility to negative emotional stimuli, and improve the Hamilton depression rating scale in the patients with depression. Repetitive TMS applied over the right dlPFC produces significant improvement in panic and depressive symptoms. Repetitive TMS treatment on the right dlPFC can also affect early stage affective visual processing, and enhance differential affective responses to fearful stimuli in individuals with anxiety. Transcranial direct current stimulation applied over the dlPFC can significantly improve the depressive symptom, cognitive function, such as working memory and attention, and stress-induced working memory deficits, and modulate the negative attentional bias in patients with depression. In depression, complicated pathogenesis, diversified pathogenic factor, manifold clinical symptoms, and individual differences make it difficult for the treatment of depression. Therefore, high-quality multicenter randomized clinical trials are required to confirm the best optimal treatment for patients with depression, for instance, the frequency of rTMS, timing and course of the treatment, and evaluation of the therapeutic effect. In addition, some studies have indicated the changes of neural excitability and the alteration of various neurochemicals may be neurobiological mechanisms of the antidepressant effect of rTMS. Notwithstanding, there are also some potential mechanisms remaining to be revealed. In addition, the short- and long-term implications of NBS in patients with depression should get sufficient attention. Besides depression, other psychiatric disorders, for instance, schizophrenia, addiction, bulimia nervosa, and posttraumatic stress disorder, have exhibited favorable responses to NBS treatment. However, NBS also needs more evidence to verify the efficacy and explore physiological basis of affective processing in these psychiatric disorders.

## Conclusion

The PFC is the most frequently studied region with NBS. The application of NBS over the PFC can significantly modulate affective processing and improve the clinical symptoms of several psychiatric disorders, such as depression and schizophrenia. The effects of TMS over the PFC on affective processing depend on the exact stimulation frequency, the number of consecutive stimulation sessions, and the targeted sub-region of the PFC. Although some potential mechanisms have been identified, the overall picture is yet to be complete. The application of TMS over the left dlPFC can increase brain activity in the left dlPFC and the anterior cingulate gyrus, whereas it also decreases brain activity measured in the left fusiform gyrus, the left cerebellum, and the right dlPFC. Transcranial magnetic stimulation applied to the right dlPFC can increase event-related neural activity bilaterally in the parietal and temporal cortex, increase early stage affective visual processing, and enhance differential affective responses to fearful stimuli. Transcranial direct current stimulation can enhance neuronal activation in the PFC, dorsal hippocampus, ventral tegmental area, and nucleus accumbens. However, the exact mechanisms underlying the tDCS effects remain unclear. As more and more studies investigate the effects and underlying mechanisms of NBS, its clinical applications should continue to be scrutinized.

## Author Contributions

WL, YL, XZ, ZC, and BL wrote the manuscript. WY and BL provided the critical revisions. All authors approved the final version of the manuscript for submission.

## Conflict of Interest Statement

The authors declare that the research was conducted in the absence of any commercial or financial relationships that could be construed as a potential conflict of interest.
